# First blood: the endothelial origins of hematopoietic progenitors

**DOI:** 10.1007/s10456-021-09783-9

**Published:** 2021-03-30

**Authors:** Giovanni Canu, Christiana Ruhrberg

**Affiliations:** UCL Institute of Ophthalmology, University College London, 11-43 Bath Street, London, EC1V 9EL UK

**Keywords:** Endothelium, EndoHT, Hematopoiesis, Erythro-myeloid progenitor, Hematopoietic stem cell, Fetal liver, Yolk sac, Tissue‐resident macrophage

## Abstract

Hematopoiesis in vertebrate embryos occurs in temporally and spatially overlapping waves in close proximity to blood vascular endothelial cells. Initially, yolk sac hematopoiesis produces primitive erythrocytes, megakaryocytes, and macrophages. Thereafter, sequential waves of definitive hematopoiesis arise from yolk sac and intraembryonic hemogenic endothelia through an endothelial-to-hematopoietic transition (EHT). During EHT, the endothelial and hematopoietic transcriptional programs are tightly co-regulated to orchestrate a shift in cell identity. In the yolk sac, EHT generates erythro-myeloid progenitors, which upon migration to the liver differentiate into fetal blood cells, including erythrocytes and tissue-resident macrophages. In the dorsal aorta, EHT produces hematopoietic stem cells, which engraft the fetal liver and then the bone marrow to sustain adult hematopoiesis. Recent studies have defined the relationship between the developing vascular and hematopoietic systems in animal models, including molecular mechanisms that drive the hemato-endothelial transcription program for EHT. Moreover, human pluripotent stem cells have enabled modeling of fetal human hematopoiesis and have begun to generate cell types of clinical interest for regenerative medicine.

## Introduction

Early in vertebrate development, mesodermal cells produce a wide range of specialized cell types, including the first vascular endothelial and blood cells as a prerequisite for embryo growth and organogenesis [1]. Work carried out in chicken, fish, and amphibian embryos provided initial information on the mesodermal production of both endothelial and blood cells [2]. Thereafter, the mouse embryo has become the organism of choice to model the connection between blood vessel growth and hematopoiesis in mammals [2]. Collectively, these animal studies have shown that the basic principles governing early hematopoietic development are largely conserved across vertebrate classes, with only a few exceptions, and therefore provided significant progress towards the ultimate aim of understanding mechanisms that drive human hemato-vascular development [2]. Here, we provide a comprehensive view of current knowledge on hemato-vascular connections, including recent findings from studies that have employed genetic lineage tracing, stem cell culture, or single cell transcriptomics to study hematopoiesis. We also compare cellular and molecular mechanisms relevant for hemato-vascular origins in mouse and human embryos, highlighting both major similarities and known differences. Further, we will discuss how culture systems based on human pluripotent stem cells can be used both to model human hematopoietic development and to generate blood cells of clinical interest.

## Ontogeny of the hematopoietic system

**Fig. 1:**
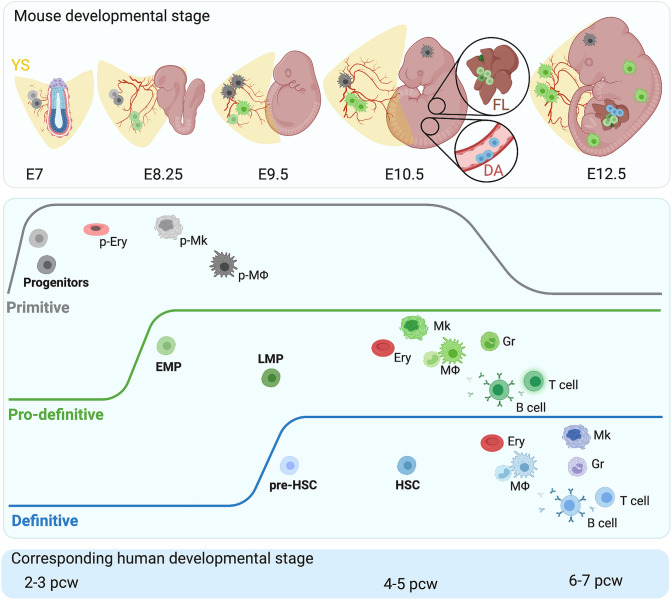
Ontogeny of the hematopoietic system. Top panel: Hematopoietic development proceeds in three spatiotemporally overlapping waves termed primitive, pro-definitive, and definitive hematopoiesis, indicated with gray, green, and blue colors, respectively. Each wave produces distinct hematopoietic progenitors, which are shown in the top panel at their site of origin and their destination in the embryo at the relevant developmental stages. Pro-definitive progenitors arising in the yolk sac (YS) and hematopoietic stem cells (HSCs) arising in the dorsal aorta (DA) co-exist in the fetal liver (FL), as shown at higher magnification for E10.5. Middle panel: Each hematopoietic wave generates a unique, essential, and complementary set of circulating and tissue-resident hematopoietic cells. The primitive wave produces erythrocytes (p-Ery), megakaryocytes (p-Mk), and macrophages (p-MΦ) that remain in the yolk sac or invade the embryo to generate microglia. The pro-definitive wave generates erythro-myeloid progenitors (EMPs) and lympho-myeloid progenitors (LMPs). The definitive wave generates pre-HSCs, which mature into HSCs capable of self-renewal. Both pro-definitive and definitive wave progenitors travel to the liver, where they produce erythrocytes (Ery), megakaryocytes (Mk), granulocytes (Gr), T cells and B cells as well as monocyte-derived macrophages (MΦ). EMP-derived MΦs colonize the embryo and constitute the majority of tissue-resident MΦs at birth. Bottom panel: Hematopoietic development is thought to follow similar principles in human, with the corresponding developmental stages shown

Early studies identified two hematopoietic waves in the mammalian embryo: an early extra-embryonic wave in the yolk sac that produces transient blood cells and was termed primitive, and an intraembryonic wave that generates hematopoietic stem cells (HSCs) termed definitive. Subsequently, the yolk sac was shown to also produce hematopoietic cells that seed the embryo and persist into fetal and, to some extent, adult life [2]. For this reason, a model of three hematopoietic waves (Fig. 1) is now widely accepted: (1) primitive hematopoiesis, which takes place in the yolk sac and produces short-lived blood cells; (2) pro-definitive hematopoiesis, which originates in the yolk sac but produces hematopoietic progenitors that seed the embryo to contribute blood cells until birth; (3) definitive hematopoiesis, which originates in the embryo and produces HSCs that initially seed the fetal liver and thereafter permanently colonize the bone marrow to support adult hematopoiesis. All three waves are spatiotemporally connected to blood vascular development.

### Primitive hematopoiesis

The first wave of blood cell production begins when vascular and hematopoietic cells differentiate from mesodermal progenitors and progressively organize themselves into blood islands in the extra-embryonic yolk sac. This process occurs in the mouse from embryonic day (E) 7.0 onwards, equivalent to the second to third week of human gestation [3] (Fig. 1).

*Ex vivo* differentiation assays suggested that mouse E7.0 blood islands contain a pool of bipotent progenitors for primitive erythrocytes and megakaryocytes as well as unipotent progenitors that differentiate into primitive macrophages [4]. In the mouse yolk sac in vivo, erythrocytes are already observed at E7.0, whereas macrophages are detected only from E9.0 onwards [5] (Fig. 1). These primitive macrophages arise from their progenitors without a monocyte intermediate [6] and either stay in the yolk sac or invade the embryo proper to give rise to the first tissue-resident macrophages, including microglia in the brain [7, 8] (Fig. 1). In contrast to their adult equivalents, primitive erythrocytes are nucleated and express embryonic-specific hemoglobin (composed in mouse of α-chains encoded by* Hba-x* and β-chains encoded by* Hbb-y*) [9, 10]. The term ‘early erythro-myeloid progenitors’ has been used in some publications to refer to primitive hematopoietic progenitors. However, we will not adopt this nomenclature here, as there is no direct evidence for a homogenous cell population that arises from mesoderm and gives rise to progenitors with dual erythroid and myeloid potential.

Due to ethical and technical challenges, limited knowledge is available about early hematopoiesis in the human embryo. Nevertheless, it has been shown that the human yolk sac at 2–3 weeks post conception also contains nucleated erythrocytes that express embryonic hemoglobins (with α-chains encoded by* HBZ* and β-chains encoded by* HBE1*), as well as some megakaryocytes and macrophages [11–13]. Moreover, the differentiation of human pluripotent stem cells allows the production of primitive erythrocytes and macrophages in vitro, implying that human primitive hematopoiesis follows similar principles as those described for the mouse [14–16].

### Pro-definitive hematopoiesis

The second, pro-definitive wave of hematopoiesis arises in the yolk sac vasculature from a subset of endothelial cells termed hemogenic endothelial cells (Fig. 1). These cells undergo an endothelial-to-hematopoietic transition (EHT), whereby a change in cellular identity causes hematopoietic progenitors to bud off the endothelium as clusters of round cells [17, 18]. This hematopoietic wave is sometimes referred to as ‘transient definitive,’ because it generates multipotent progenitors that can only reconstitute hematopoiesis transiently after transplantation into a bone marrow-ablated adult mouse.

In the mouse, pro-definitive hematopoiesis begins at E8.25 to produce *bone fide* erythro-myeloid progenitors (EMPs) with a dual erythroid and myeloid fate [19, 20] (Fig. 1). With the establishment of the blood circulation by E10.5, EMPs are transported out of the yolk sac and start colonizing the fetal liver, from where they sustain hematopoiesis until birth. In the liver, EMPs differentiate into multiple types of blood cells, including megakaryocytes, enucleated erythrocytes with fetal-type hemoglobin β-chains (Hbb-bh1 in the mouse and HBG1 and HBG2 in humans) [9, 10], and monocytes that infiltrate other organs via the circulation to generate tissue-resident macrophages [5] (Fig. 1). From E12.5 onwards, these monocyte-derived macrophages gradually replace those produced during the primitive wave, and EMP-derived macrophages are now thought to constitute the majority of tissue-resident macrophages found at birth [19–22] (Fig. 1). The brain is an exception, because microglia of primitive macrophage origin are retained in this organ as a stable and self-renewing population, possibly protected by the blood–brain barrier from replacement with later-born, monocyte-derived macrophages [5, 7, 8] (Fig. 1). Furthermore, EMPs generate granulocytes, a cell type that is not produced during primitive hematopoiesis [23] (Fig. 1).

In the mouse yolk sac from E9.5 onwards, pro-definitive hematopoiesis also produces lymphoid-restricted progenitors and multilineage lympho-myeloid progenitors [24]. Like EMPs, these cells are thought to bud from the yolk sac hemogenic endothelium and home to the fetal liver (Fig. 1). It remains, however, unclear whether EMPs, lymphoid-restricted progenitors, and lympho-myeloid progenitors emerge sequentially from a common pool or distinct subsets of hemogenic endothelial cells [25]. In the liver, the lymphoid progenitors differentiate into circulating B and T cells as well as tissue-resident, innate lymphocytes in multiple organs, including γδ T cells in the skin, lung, and liver; B1a cells in the gut and lung; and marginal zone B cells in the spleen [26–28]. Similar to microglia, innate lymphocytes are thought to be exclusively produced during embryonic development and persist as self-renewing cells into adulthood; accordingly, when depleted in the adult, a subset of these cells is not replaced after HSC transplantation [29, 30].

Although human pro-definitive progenitors have not been characterized, the human fetal liver is seeded by growing numbers of CD34+/CD45 + cells from the third to fourth week of gestation, prior to HSC emergence [6]. These cells likely represent hematopoietic cells akin to mouse yolk sac-derived EMPs and lymphoid progenitors; it is therefore usually accepted that cells equivalent to mouse pro-definitive progenitors must exist in humans [31].

### Definitive hematopoiesis

The third, definitive hematopoietic wave arises intraembryonically from hemogenic endothelial cells in the aorta-gonad-mesonephros (AGM) region between E9.5 and E11.5 in mouse and between the fourth and fifth post-conception week in human [32–34] (Fig. 1). These hemogenic endothelial cells upregulate the transcription factor RUNX1, which is thought to coordinate the activation of the hematopoietic transcriptional program that drives EHT [35]. EHT manifests as the budding of intra-aortic hematopoietic cell clusters from the ventral endothelium in the dorsal aorta in both mouse and human (Fig. 1). Such clusters also bud to a minor extent from the dorsal endothelium in the mouse aorta [36]. The first hematopoietic stem cells (HSCs) with robust long-term multilineage reconstitution potential are detected in the mouse AGM from E10.5 onwards [31, 34] (Fig. 1). These HSCs enter the circulation to seed the fetal liver, where they undergo expansion and further maturation [34, 37]. Eventually, HSCs exit the liver to colonize the bone marrow to sustain life-long hematopoiesis after birth [38]. In the human embryo, the first HSCs are detected at 4 to 5 weeks of gestation, when they can be identified based on their ability to reconstitute hematopoiesis in a mouse host [31, 34].

Prior to the emergence of HSCs, from E9.5 onwards in the mouse, the AGM generates hematopoietic progenitors with blood clonogenic activity and multilineage differentiation potential in vitro, but lacking long-term reconstitution upon transplantation into a bone marrow-ablated adult [37]. As long-term reconstitution is a hallmark of HSCs, these cells are considered to be HSC precursors and termed pre-HSCs (Fig. 1). Accordingly, AGM hematopoiesis has been suggested to be a multistep process in which the hemogenic endothelium first produces pre-HSCs that lack stem cell activity before producing *bone fide* HSCs [37]. The sequential expression of the cell surface markers VE-Cadherin (CDH5), CD41 (ITGA2B), CD43 (SPN), and CD45 (PTPRC) has been used to discern consecutive maturation stages of these HSC precursors into pro-HSCs, pre-HSCs type I, and pre-HSCs type II. These precursors are thought to mature within the intra-aortic clusters into HSCs, which then travel through the circulation to the liver. Additionally, it has been suggested that some pro- and pre-HSCs home to the liver to complete their maturation [39].

Although pro- and pre-HSCs have thus far only been defined in the mouse, the human AGM also produces blood clonogenic cells without the capability of long-term engraftment prior to *bona fide* HSC emergence [40, 41]. Moreover, in vitro differentiation of human pluripotent stem cells yields hematopoietic progenitors with multilineage differentiation potential, but no stem cell activity [42, 43], and the transcriptional signature of these in vitro-derived hematopoietic progenitors is similar to that of hematopoietic cells in the human AGM in vivo [44]. Altogether, these findings suggest that hematopoietic precursors similar to mouse pro-HSCs and pre-HSCs exist in human before mature HSCs are produced.

Secondary hematopoietic sites have also been reported to produce HSCs immediately after the onset of AGM hematopoiesis, both in mouse and human embryos. Specifically, EHT occurs in the placental, vitelline, and umbilical arteries as well as in the arteries in the embryonic head to produce hematopoietic progenitors that colonize the liver and contribute to the fetal HSC pool [45–48]. Importantly, the presence of pro-definitive progenitors in the liver temporally overlaps with that of HSCs derived from the AGM and secondary hematopoietic sites. Thus, both EMPs, lymphoid progenitors, and HSCs contribute to blood cell production via the fetal liver. Being populated by hematopoietic progenitors from various sites, the liver gradually becomes the main hematopoietic organ from E12.5 in the mouse, and the sixth to seventh post-conception week in humans. In fact, the liver remains the most important site for blood cell production until around birth, when HSCs seed the bone marrow.

Notably, HSC contribution to monocyte-derived tissue macrophages appears to be minimal at least until birth in mice [5, 19, 21, 24]. Thereafter, bone marrow-derived monocytes are recruited into organs to promote innate immunity, but whether they contribute to the pool of tissue-resident macrophages remains unclear. In fact, it has been suggested that EMP-derived tissue-resident macrophages self-renew and thus are maintained into adulthood under physiological conditions. By contrast, the normal pool of EMP-derived macrophages appears to be complemented or replaced by bone marrow HSC-derived monocytes after irradiation, in disease or with aging [49–54]. Accordingly, much research is currently directed at determining the relative contribution of EMPs and HSCs to adult tissue-resident macrophages in various organs under steady state conditions.

## Molecular mechanisms for hematopoietic specification

### Key transcription factors cooperate to induce hematopoietic specification

The transcription factor RUNX1 is expressed in hemogenic endothelial cells in the AGM at the time when hematopoietic clusters emerge [35] and enables HSC production in the AGM and other secondary hematopoietic sites [55, 56]. In addition, RUNX1 is required for the generation of EMPs in the yolk sac [57, 58]. These findings suggest a similar requirement for RUNX1 during EHT at distinct sites, i.e., for the production of both EMPs and HSCs. Loss of RUNX1 in the mouse causes lethality at E12.5 with severe anemia due to the absence of pro-definitive and definitive blood cells [58, 59]. In contrast, RUNX1 is dispensable for primitive hematopoiesis, possibly because this process does not involve EHT; nevertheless, the maturation of primitive blood cells appears abnormal in the absence of RUNX1 [58, 59].

During EHT, RUNX1 acts in concert with other key transcription factors such as TAL1 and GATA2. In the embryo, TAL1 is necessary for the specification of all three hematopoietic waves [60, 61], whereas in the adult, TAL1 is required for the maintenance of HSCs and hematopoietic progenitors as well as for blood lineage commitment [62]. TAL1 action is indeed strongly context-dependent, as it forms complexes with other hematopoietic transcription factors; thus, TAL1 regulates HSC maintenance with GATA2 but directs erythroid and megakaryocytic differentiation with GATA1 [60–62]. GATA2 is required for both pro-definitive and definitive hematopoiesis, and mutant mouse embryos lacking GATA2 die before E11.5 due to severe anemia [63]. By contrast, GATA2 is not strictly required for primitive hematopoiesis, when the main factors driving primitive erythropoiesis are GATA1 and TAL1 [64].

### Several signalling pathways cooperate to induce EHT

Although little is still known about the molecular mechanisms that induce the specification of hemogenic endothelium and therefore EHT in the yolk sac, multiple signalling pathways have been implicated in these processes during AGM hematopoiesis. Specifically, it was shown that the aortic endothelium receives various morphogenetic cues along the dorsoventral axis, including regulators of the BMP and WNT signalling pathways, with additional roles for cell–cell contact-dependent notch signalling and cell cycle regulation. It has also been proposed that the different origins of ventral and dorsal aortic endothelial cells from lateral plate and paraxial mesoderm, respectively, may contribute to the dorsoventral polarization of hematopoiesis in the aorta [65, 66]. Below, we discuss key findings made in the mouse embryo.

The BMP pathway helps establish the HSC niche in the AGM region, with BMP signalling largely restricted to the ventral side of the AGM, where the subaortic mesenchyme produces BMP4 [67]. BMP signaling is further modulated by FGF, which is produced by the dorsal somitic tissue and represses BMP4 transcription and induces BMP inhibitors such as noggin [68]. As a result, HSC emergence is spatially restricted to the ventral portion of the aorta. Importantly, BMP signaling in the AGM is transient to enable the maturation of budding cells into functional HSCs [69]. Thus, soon after BMP activation, the morphogen SHH induces ventral noggin expression, which then suppresses BMP signaling [70, 71]. Furthermore, the ventrally localized BMPER initially activates the BMP pathway, but thereafter acts as an inhibitor once expression reaches a threshold level [72]. Accordingly, localized and transient BMP signalling is a prerequisite for correct spatiotemporal patterning of HSC emergence in the AGM [69, 73]. Canonical WNT signaling is also necessary for HSC specification in the AGM, but dispensable for the subsequent maintenance of emerging HSCs [74]. Retinoic acid appears to ensure that WNT signaling is inhibited in emerging hematopoietic cells, so that WNT activation is restricted to the endothelium [75].

Cooperating with BMP and WNT signaling in the AGM, cell–cell contact-dependent notch activation promotes hemogenic specification. Specifically, DLL4/NOTCH1 signaling activates the arterial program while JAG1/NOTCH1 signaling blocks it to induce hemogenic specification [76]. Although both DLL4 and JAG1 are expressed in the dorsal aorta, JAG1 has a higher affinity for the NOTCH1 receptor and results in a lower signaling strength that helps induce hemogenic identity [76]. In the yolk sac, transcription of *Alox5* and *Alox5ap*, encoding for proteins with a central role in leukotriene production, are upregulated in hemogenic endothelial cells at E8.5 and shown to be functionally important [77].

Downstream of extrinsic signals, cell cycle regulation has emerged as a key player in orchestrating hemogenic specification and EHT. In the yolk sac, retinoic acid-dependent notch activation mediates cell cycle arrest to create permissive conditions for endothelial cells to become hemogenic [78]. In the AGM, the anatomical position of emerging progenitors within hematopoietic clusters correlates with progressive cell cycle activation, whereby slowly cycling cells are frequently found at the base of the cluster in association with the underlying endothelium, while rapidly cycling cells are located at apical positions within the cluster [37]. Most HSCs in the fetal liver are actively cycling, possibly to expand the stem cell pool and rapidly produce blood cells; by contrast, HSCs adopt a quiescent phenotype later during development and upon seeding the bone marrow [79].

Genetic studies have highlighted the importance of cell cycle regulators, especially cyclins and CDKs, for early hematopoietic development. Mice deficient in the three cyclin D genes CCND1, CCND2, and CCND3 die during late embryogenesis with severe hematopoietic defects; these include a reduced number of hematopoietic progenitors and HSCs in the liver, which accumulate in the G1 phase of the cell cycle, and only a few red blood cells in the circulation [80]. Hematopoietic progenitors from these knockout mice are also unable to provide even short-term reconstitution upon transplantation. Further, loss of both CDK4 and CDK6, which bind to the cyclin Ds to drive G1 phase progression, causes late embryonic lethality with defective fetal hematopoiesis, similar to mice lacking the cyclin D genes [81].

In the mouse, the adult bone marrow niche actively maintains HSC quiescence, which is believed to contribute to HSC longevity, at least in part by minimizing cellular stress due to repeated DNA replication and extensive cellular metabolism [82]. HSCs in the human bone marrow also adopt a quiescent cell cycle state. Exit from quiescence to produce blood cells is tightly regulated, and two HSC subsets have been defined by their propensity to enter cell cycle progression; the so-called ‘short-term HSCs’ express CDK6 and immediately re-enter the cell cycle upon mitogenic stimulation, whereas ‘long-term HSCs’ delay cell cycle entry by 5–6 h [83]. Delayed cell cycle entry in long-term HSCs is caused by the absence of CDK6, which needs to be expressed to exit the quiescent state [83]. The differential expression of CDK6 in the two HSC subsets may represent a safeguarding control mechanism to preserve the HSC pool by ensuring that long-term HSCs only exit quiescence upon sustained exposure to proliferation and differentiation signals that are necessary for CDK6 expression, while short-term HSCs are primed to quickly respond to hematopoietic demand. In contrast to knowledge in the adult human bone marrow, limited information is currently available for cell cycle regulation and signalling pathways in human hematopoietic development. Nevertheless, insights into cell cycle regulation as well as key mechanisms driving human hematopoietic development have been obtained using suitable in vitro systems, which we discuss below.

## Pluripotent stem cells to model human hematopoietic development

### Investigating hemogenic specification in vitro

Whereas hematopoiesis is readily studied in model organisms, investigating hematopoiesis during human development poses technical and ethical challenges. Although tissues from deceased human embryos can be examined, the developmental stages available for research are limited, with nearly no access to samples before the fourth post-conception week. Moreover, lineage tracing studies are impossible in species other than model organisms. In this context, the in vitro differentiation of human pluripotent stem cells (hPSCs) provides a valuable tool to recapitulate the earliest stages of human hematopoietic development. This approach has allowed, for example, to elucidate signalling pathways required for human primitive vs. definitive hematopoiesis [14, 15] and the importance of cell cycle entry as a necessary condition for EHT [44]. Moreover, hPSC systems allow hematopoietic disease modeling and provide a useful resource for regenerative medicine.

**Fig. 2:**
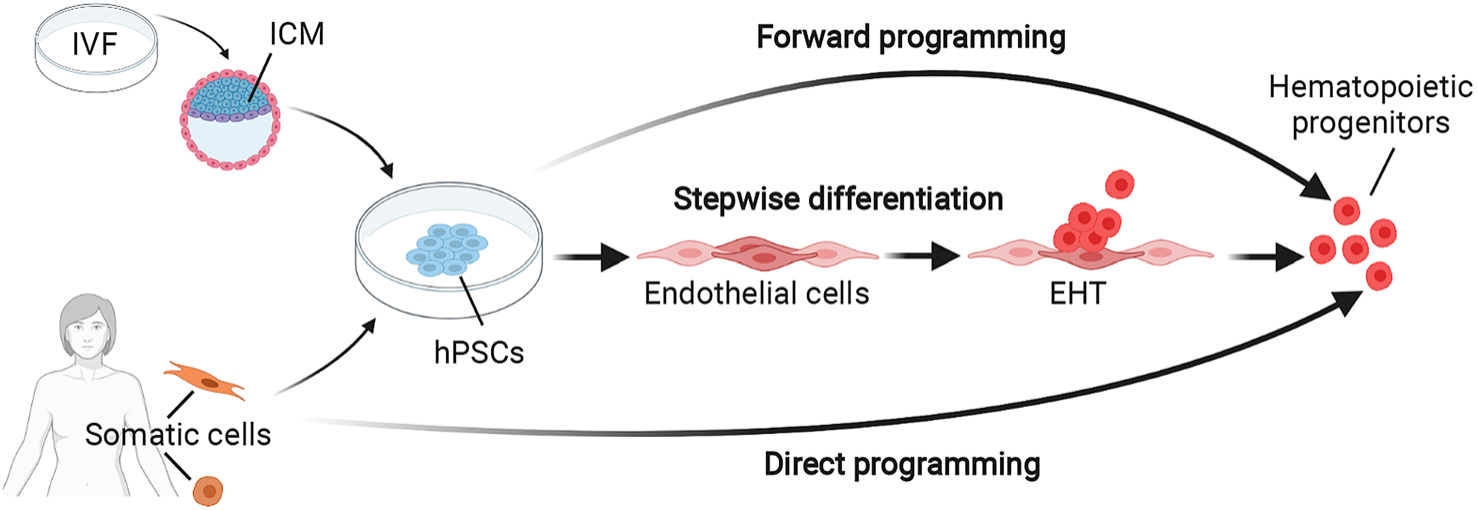
Strategies for hematopoietic cell production in vitro. Human pluripotent stem cells (hPSCs), including embryonic stem cells and induced pluripotent stem cells, have been used to model human hematopoiesis in vitro and to generate differentiated blood cells. Embryonic stem cells are derived from the inner cell mass (ICM) of donated, surplus human pre-implantation embryos generated for in vitro fertilization (IVF). Induced pluripotent stem cells are derived from reprogrammed adult somatic cells, usually fibroblasts or circulating blood cells. Both types of hPSCs are able to self-renew in appropriate culture conditions and can be induced to differentiate into hematopoietic cells. Forward programming (top): The enforced expression of several key transcription factors activates the hematopoietic program that converts hPSCs into hematopoietic cells. Stepwise differentiation (middle): The sequential administration of cytokines and small molecules induces the developmental steps that drive hematopoietic differentiation in vivo. Direct programming (bottom): The enforced expression of several key transcription factors in adult somatic cells converts them directly into hematopoietic cells

The two types of hPSCs used to date include embryonic stem cells derived from the inner cell mass of the pre-implantation embryo and induced pluripotent stem cells derived from reprogramming of adult somatic cells [84–86] (Fig. 2). Both cell types retain the ability to grow almost indefinitely under suitable culture conditions but can be induced to differentiate into hematopoietic cells with multiple blood cell potential [87, 88]. The traditional approach to induce hematopoiesis from hPSCs involves the stepwise differentiation into mesoderm and then endothelium, including hemogenic endothelium that undergoes EHT (Fig. 2). Alternatively, the enforced expression of specific combinations of transcription factors that induce the hematopoietic program, either in hPSCs (forward programming) or in somatic cells (direct programming), can produce blood cells [89, 90] (Fig. 2). Forward programming has been used, for example, to convert hPSCs directly into megakaryocytes to produce platelets for transfusion medicine [91]. Nonetheless, several challenges remain, in particular, a low throughput of the process and the ability to ensure full functionality of the product in vivo.

Induced pluripotent stem cells are of particular interest for regenerative medicine, as they can be obtained by reprogramming somatic cells from adult human donors and might therefore enable therapeutic production of patient-specific, immunocompatible HSCs. In addition, comparing such cells from patients with specific genetic diseases with isogenic cell lines in which the underlining mutation has been corrected can provide knowledge of the molecular mechanisms that determines disease. Ultimately, the genetic correction of donor cells carrying detrimental mutations may enable personalized cell therapies.

Below, we review how key signalling pathways and transcription factors driving hematopoietic specification *in vivo* are harnessed for differentiating or reprogramming hPSCs into blood cells.

### Differentiating human pluripotent stem cells towards a hematopoietic fate

**Fig. 3:**
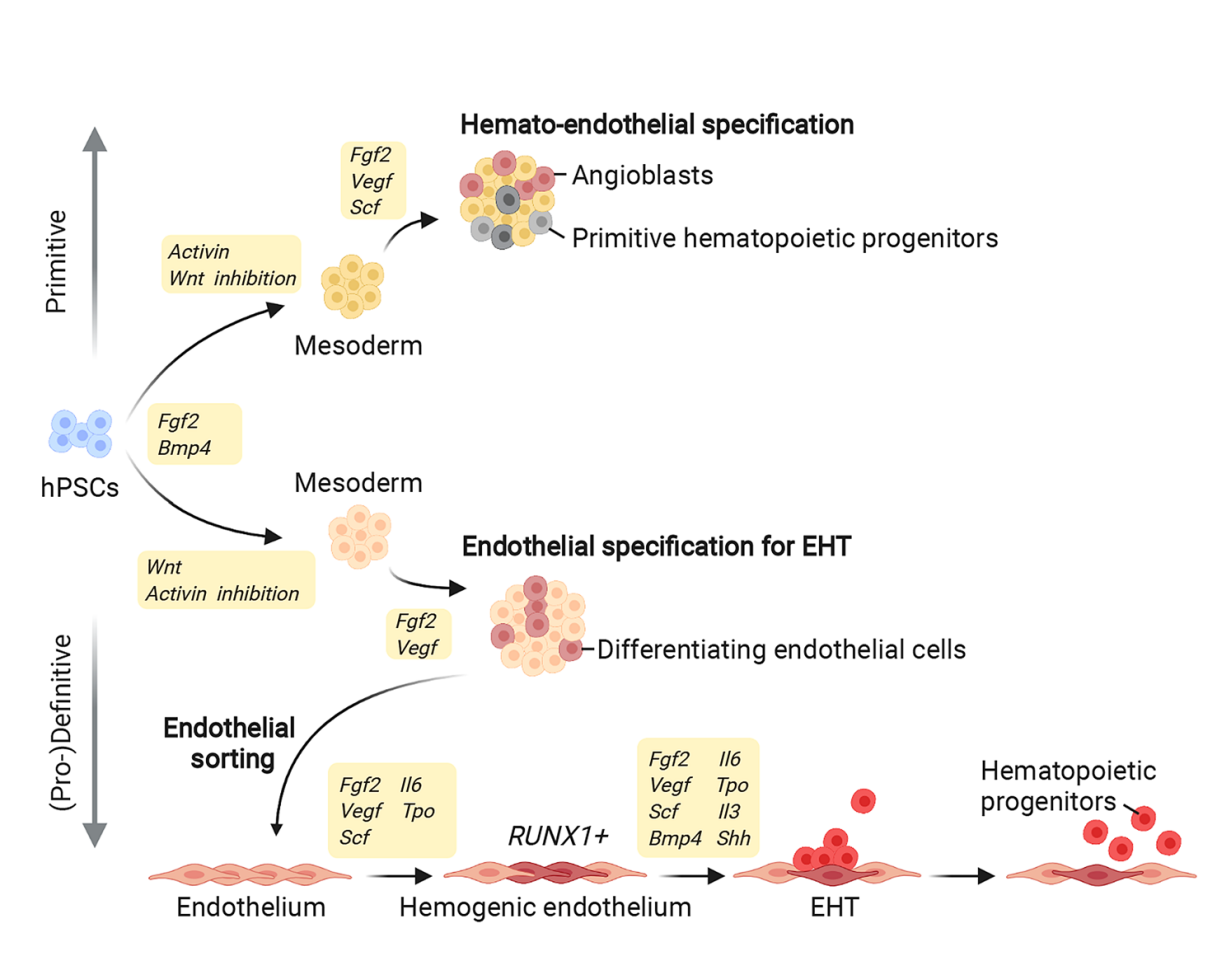
Induction of distinct hematopoietic waves using hPSCs. Modulating key signalling pathways early during stepwise hPSC differentiation enables the production of primitive versus (pro-)definitive hematopoietic cells (only key steps shared between various different protocols are shown). FGF and BMP induce hPSC differentiation towards mesoderm. When combined with activin activation and WNT inhibition, mesodermal cells differentiate further into primitive hematopoietic cells. Instead, WNT activation with activin inhibition induces mesodermal cells to differentiate further into endothelial cells, including hemogenic endothelial cells that express RUNX1 and undergo EHT to produce (pro-)definitive hematopoietic cells

Initially, hPSCs were cultured and differentiated in serum-supplemented media with feeder cells to model the microenvironment driving differentiation in vivo. However, these poorly refined culture conditions did not allow efficient or homogeneous differentiation along specific lineages and were therefore gradually replaced by more refined culture systems with carefully controlled stepwise differentiation [92, 93] (Figs. 2 and 3). Accordingly, most culture methods now use serum- and feeder-free systems based on chemically defined culture media, supplemented with recombinant cytokines and small molecules administered in a precise temporal order to induce differentiation (Fig. 3). This approach is designed to mimic the in vivo signals that sequentially control pluripotency, early germ layer induction, hemogenic specification, and finally endothelial-to-hematopoietic transition [15, 16, 42–44, 94]. In fact, using hPSCs for blood cell production requires accurate control over maintaining the pluripotent state versus subsequently inducing differentiation to recapitulate the distinct stages of hematopoietic development. Firstly, regulating the signalling pathways that maintain the undifferentiated pluripotent state is important, because suboptimal pluripotency conditions can thereafter affect differentiation quality [86, 92]. Secondly, to induce exit from pluripotency and germ layer specification towards endoderm, mesoderm, or neuroectoderm, several signalling pathways need to be coordinately modulated [95–97]. Thirdly, mesoderm needs to be induced to activate the hemato-endothelial transcriptional program as a prerequisite for specification of hemogenic endothelial cells, EHT, and thus production of pro-definitive and/or definitive, rather than primitive, blood cells (Fig. 3).

Understanding the molecular mechanisms that drive each of these distinct stages in vivo is fundamental to recapitulate the progression of developmental hematopoiesis in vitro. Early studies with hPSCs showed that the FGF and activin/nodal signalling pathways are master gatekeepers of pluripotency [86, 98], but that they also regulate germ layer specification [95, 96]. This dual role of FGF and activin/nodal signalling depends on crosstalk with other key signalling pathways, such as the BMP and WNT pathways. In particular, the functional interaction between these and other pathways serves to recreate signals that in vivo convey the position of cells within the embryo along the anteroposterior axis, where multiple morphogenetic gradients of agonists and inhibitors evoke position-dependent fate decisions [97, 99]. Thus, manipulating the relative activation levels of core signalling pathways such as FGF, activin/nodal, BMP, and WNT allows proper germ layer specification and subsequent germ layer patterning in hPSC cultures [95, 96, 100–102] (Fig. 3). Despite these advances, recapitulating the three distinct hematopoietic waves using hPSC differentiation methods remains a considerable challenge.

### Inducing sequential hematopoietic waves in human pluripotent stem cells

Several protocols have achieved hPSC differentiation into hemogenic endothelial cells capable of undergoing EHT [14, 16, 31, 42–44, 87, 94] (Fig. 3). Although early culture methods produced a mixture of primitive and definitive blood cells, we now have protocols that impair the production of primitive progenitors and generate endothelial cells that undergo EHT to produce more mature (pro-)definitive hematopoietic progenitors [43]. In these protocols, the mesoderm stage of a core hPSC differentiation protocol is manipulated to produce either primitive or definitive hematopoietic progenitors [15, 42] (Fig. 3). Specifically, the inhibition of the WNT canonical pathway, when combined with activin induction, enriches for hematopoietic progenitors of the primitive wave; conversely, the induction of WNT signalling, in the absence of activin induction, enriches for hematopoietic progenitors of the definitive wave (Fig. 3). To develop these methods, functional properties like T lymphocyte potential and production of erythrocytes expressing fetal-type hemoglobin have been used as hallmarks of definitive hematopoiesis [15, 42]. Yet, despite excluding primitive progenitors, these criteria are not sufficient to discriminate between the pro-definitive and definitive hematopoietic lineages [20, 24, 33, 34]. Therefore, distinguishing molecular markers for the two waves of hematopoietic progenitors are needed. Recent mouse work proposed HLF as a marker that distinguishes HSCs from EMPs, at least in mouse [103], and its usefulness as a marker for human hematopoiesis should therefore be investigated.

Current hPSC differentiation systems cannot produce cells capable of long-term multilineage reconstitution upon transplantation, suggesting that *bona fide* HSCs are not generated. Nevertheless, a high transcriptional similarity of hPSC-derived hematopoietic progenitors to human AGM-derived hematopoietic cells [44] raises the possibility that the rate limiting step in these culture methods is currently not a lack of AGM-like EHT, but the incomplete maturation of precursor cells into mature HSCs. An improved understanding of the molecular and cellular mechanisms that coordinate EHT with downstream HSC maturation steps will therefore be pivotal to generate mature HSC-like cells that may be used in regenerative medicine or disease modeling. In the meantime, the differentiation of hPSCs has provided a convenient platform for mechanistic studies of hemogenic endothelial cells, EHT, and hematopoietic differentiation under controlled conditions.

### Advances in understanding mechanisms controlling EHT using in vitro systems

Culture models based on hPSC differentiation have demonstrated the importance of RUNX1 for EHT via suppression of the endothelial and activation of the hematopoietic transcriptional program [104] (Fig. 3). Thus, RUNX1 activates the downstream transcription factors GFI1 and GFI1B, which then repress endothelial identity by downregulating core endothelial genes such as CDH5 and TIE2 [105–107]. Concomitantly, RUNX1 cooperates with TAL1 and GATA2 to activate the expression of hematopoietic genes [108, 109]. Further, in vitro work showed that TAL1 inhibits cardiac lineage specification in early mesoderm, inducing the differentiation of lateral plate mesoderm towards hematopoietic and endothelial fates and subsequently consolidating the hematopoietic fate [110]. Moreover, the leukotriene C4 increased, but the Alox5 inhibitor Zileuton reduced, hematopoietic colony numbers during the in vitro differentiation of murine embryonic stem cells, demonstrating that the upregulation of *Alox5* and leukotriene production are functionally important and confirming what was shown for mouse yolk sac hemogenic endothelial cells [77]. Consistent with prior work in the mouse embryo [37], hPSC modeling of human EHT corroborated that cell cycle regulation by specific cyclin-CDK complexes modulates the timely activation of the hematopoietic transcriptional network [44]. Importantly, hPSC-derived hemogenic endothelial cells possess a quiescent cell cycle profile, being enriched in the G0/G1 phase, which is thought to represent a time window during which cells are receptive to extracellular cues and can undertake cell fate decision by changing gene expression program [111]. Further, hemogenic endothelial cells need to re-activate the cell cycle in order to undergo EHT and produce hematopoietic cells. Accordingly, a transient cell cycle block during EHT causes hemogenic endothelial cells to downregulate RUNX1 and permanently lose their hemogenic potential to retain a non-hemogenic endothelial cell identity [44]. Thus, dynamic cell cycle control appears necessary for hemogenic endothelial specification and EHT.

### Hematopoietic reprogramming yields HSC-like cells

As stepwise hPSC differentiation has not yet yielded mature HSCs capable of engrafting a host upon transplantation, alternative reprogramming methods have been developed in parallel to produce HSC-like cells or HSC progeny (Fig. 2). A recent approach has converted mouse adult vascular endothelial cells into HSC-like cells by viral transduction with the transcription factors FOSB, GFI1, RUNX1, and SPI1, followed by co-culture with modified human umbilical vein endothelial cells [112]. In another study, human HSC-like cells were generated through a combination of hPSC stepwise differentiation and direct programming [113]. For this approach, conventional cytokine methods were used to differentiate hPSCs into hemogenic endothelial cells, which were then transduced with the seven transcription factors ERG, HOXA5, HOXA9, HOXA10, LCOR, RUNX1, and SPI. In both studies, the resulting HSC-like cells were capable of multilineage engraftment in primary and secondary mouse recipients. These results have advanced our knowledge of the molecular requirements for the generation of HSC-like cells in vitro and have also provided proof of principle for the importance of an endothelial intermediate for HSC specification. Unfortunately, however, none of the currently available reprogramming methods are amenable to clinical application due to inherent difficulties in scaling up cell production and safety concerns associated with viral gene transduction.

## Plasticity of the hemato-vascular interaction

The hemogenic program in endothelial cells might be reversible and partially plastic. Firstly, reversible fate can be observed in vitro, because preventing EHT in hPSC-derived hemogenic endothelial cells switches off the hematopoietic program and cells instead continue to grow as non-hemogenic endothelial cells [44]. Secondly, hPSC-derived hemogenic endothelial cells appear to generate both hematopoietic and mesenchymal cells, with the two fates possibly representing alternative cell fate choices of a common progenitor, dependent on exposure to specific differentiation factors [44]. In vivo studies also suggest that EHT-derived cells can revert to an endothelial identity after passing through the circulation. Initially, viral transduction studies in quail embryos were used to show that yolk sac-derived cells can travel via the circulation into the embryo proper to form endothelial cells [114]. Thereafter, genetic lineage tracing of yolk sac endothelium showed that cells with characteristics akin to EMPs can migrate into the embryo to contribute both endothelial and mesenchymal cells to the AGM region [115]. More recently, genetic lineage tracing of hemogenic endothelium and EMPs combined with *ex vivo* culture of EMPs isolated from the blood or liver showed that EMPs can re-differentiate into endothelial cells that contribute to the vasculature of intraembryonic organs [18]. Notably, reversible and plastic fates have also been proposed for adult bone marrow-derived endothelial progenitors sharing similarities with HSCs [116], but remain controversial and have been discounted as contributors to endothelium in liver regeneration [117].

## Conclusions and future directions

The in vivo and in vitro studies described here have together provided extensive knowledge of molecular and cellular mechanisms that govern developmental hematopoiesis. Together, these studies portray complex interactions between the developing vascular and hematopoietic systems in both mouse and human. They show that multiple signalling pathways and transcription factors induce the formation of hemogenic endothelia in the yolk sac and AGM to produce pro-definitive progenitors and HSCs, respectively. While EMPs and lympho-myeloid progenitors produced in the yolk sac arrive first in the liver, they are later joined by fetal HSCs. Together, both types of progenitors sustain blood cell production until birth, after which HSCs sustain life-long hematopoiesis. Importantly, EMP-derived, tissue-resident macrophages help establish niches for definitive EHT and hematopoietic maturation leading to HSC production [118]. However, it is not yet understood how hemogenic endothelia in distinct spatiotemporal contexts might function to generate either EMPs and lympho-myeloid progenitors or instead HSCs. It is conceivable that hemogenic endothelial cells possess intrinsic differentiation potential depending on their site and time of origin and are therefore pre-specified to the type of progenitors they can produce. Alternatively, hemogenic endothelia at different locations might be functionally similar but produce different progenitors depending on external cues in their specific microenvironment. Thus, future work should address whether differences between pro-definitive and definitive hematopoiesis arise at the level of endothelial cell specification or depend on specific environmental niches. This knowledge, in turn, will provide vital information to further improve hPSC culture systems and thus enhance our ability to produce blood products of clinical interest, including short-lived blood cells for transfusion medicine, HSC-like cells with long-term multilineage reconstitution potential, and possibly progenitors capable of endothelial cell differentiation to treat ischemic diseases.

## Abbreviations

AGM: Aorta-gonad-mesonephros
DA: Dorsal aorta
FL: Fetal liver
E: Embryonic day
EHT: Endothelial-to-hematopoietic transition
EMPs: Erythro-myeloid progenitors
Ery: Erythrocyte
Gr: Granulocyte
ICM: Inner cell mass
IVF: In vitro fertilization
hPSC: Human pluripotent stem cell
HSC: Hematopoietic stem cells
LMP: Lympho-myeloid progenitors
Mk: Megakaryocyte
MΦ: Macrophage
p-Ery: Primitive erythrocyte
p-Mk: Primitive megakaryocyte
p-MΦ: Primitive macrophage
YS: Yolk sac

## References

[1] Goldie LC, Nix MK, Hirschi KK (2008). Embryonic vasculogenesis and hematopoietic specification. Organogenesis.

[2] Gritz E, Hirschi KK (2016). Specification and function of hemogenic endothelium during embryogenesis. Cell Mol Life Sci.

[3] Ferkowicz MJ, Yoder MC (2005). Blood island formation: longstanding observations and modern interpretations. Exp Hematol.

[4] Palis J, Robertson S, Kennedy M (1999). Development of erythroid and myeloid progenitors in the yolk sac and embryo proper of the mouse. Development.

[5] Hoeffel G, Ginhoux F (2018). Fetal monocytes and the origins of tissue-resident macrophages. Cell Immunol.

[6] Tavian M, Hallais MF, Peault B (1999). Emergence of intraembryonic hematopoietic precursors in the pre-liver human embryo. Development.

[7] Ginhoux F, Greter M, Leboeuf M (2010). Fate mapping analysis reveals that adult microglia derive from primitive macrophages. Science.

[8] Ginhoux F, Lim S, Hoeffel G (2013). Origin and differentiation of microglia. Front Cell Neurosci.

[9] Sankaran VG, Orkin SH (2013). The switch from fetal to adult hemoglobin. Cold Spring Harb Perspect Med.

[10] Dzierzak E, Philipsen S (2013). Erythropoiesis: development and differentiation. Cold Spring Harb Perspect Med.

[11] Bloom W, Bartelmez GW (1940). Hematopoiesis in young human embryos. Am J Anat.

[12] Fukuda T (1973). Fetal hemopoiesis - I. Electron microscopic studies on human yolk sac hemopoiesis. Virchows Arch B Cell Pathol Zell-pathologie.

[13] Luckett WP (1978). Origin and differentiation of the yolk sac and extraembryonic mesoderm in presomite human and rhesus monkey embryos. Am J Anat.

[14] Kennedy M, D’Souza SL, Lynch-Kattman M (2007). Development of the hemangioblastdefines the onset of hematopoiesis in human ES cell differentiation cultures. Blood.

[15] Sturgeon CM, Ditadi A, Awong G (2014). Wnt signaling controls the specification of definitive and primitive hematopoiesis from human pluripotent stem cells. Nat Biotechnol.

[16] Choi KD, Vodyanik MA, Togarrati PP (2012). Identification of the hemogenic endothelial progenitor and its direct precursor in human pluripotent stem cell differentiation cultures. Cell Rep.

[17] Kasaai B, Caolo V, Peacock HM (2017). Erythro-myeloid progenitors can differentiate from endothelial cells and modulate embryonic vascular remodeling. Sci Rep.

[18] Plein A, Fantin A, Denti L (2018). Erythro-myeloid progenitors contribute endothelial cells to blood vessels. Nature.

[19] Hoeffel G, Chen J, Lavin Y (2015). C-Myb + erythro-myeloid progenitor-derived fetal monocytes give rise to adult tissue-resident macrophages. Immunity.

[20] Frame JM, McGrath KE, Palis J (2013). Erythro-myeloid progenitors: “Definitive” hematopoiesis in the conceptus prior to the emergence of hematopoietic stem cells. Blood Cells Mol Dis.

[21] Gomez Perdiguero E, Klapproth K, Schulz C (2015). Tissue-resident macrophages originate from yolk-sac-derived erythro-myeloid progenitors. Nature.

[22] Ginhoux F, Guilliams M (2016). Tissue-resident macrophage ontogeny and homeostasis. Immunity.

[23] McGrath KE, Frame JM, Fegan KH (2015). Distinct sources of hematopoietic progenitors emerge before HSCs and provide functional blood cells in the mammalian embryo. Cell Rep.

[24] Böiers C, Carrelha J, Lutteropp M (2013). Lymphomyeloid contribution of an immune-restricted progenitor emerging prior to definitive hematopoietic stem cells. Cell Stem Cell.

[25] Hadland B, Yoshimoto M (2018). Many layers of embryonic hematopoiesis: new insights into B-cell ontogeny and the origin of hematopoietic stem cells. Exp Hematol.

[26] Haas JD, Ravens S, Düber S (2012). Development of interleukin-17-producing γδ T cells is restricted to a functional embryonic wave. Immunity.

[27] Kobayashi M, Shelley WC, Seo W (2014). Functional B-1 progenitor cells are present in the hematopoietic stem cell-deficient embryo and depend on Cbfβ for their development. Proc Natl Acad Sci USA.

[28] Yoshimoto M, Montecino-Rodriguez E, Ferkowicz MJ (2011). Embryonic day 9 yolk sac and intra-embryonic hemogenic endothelium independently generate a B-1 and marginal zone progenitor lacking B-2 potential. Proc Natl Acad Sci USA.

[29] Kristiansen TA, Jaensson Gyllenbäck E, Zriwil A (2016). Cellular barcoding links B-1a B cell potential to a fetal hematopoietic stem cell state at the single-cell level. Immunity.

[30] Ghosn E, Yoshimoto M, Nakauchi H (2019). Hematopoietic stem cell-independent hematopoiesis and the origins of innate-like B lymphocytes. Development.

[31] Ivanovs A, Rybtsov S, Ng ES (2017). Human haematopoietic stem cell development: from the embryo to the dish. Development.

[32] Tavian M, Coulombel L, Luton D (1996). Aorta-associated CD34 + hematopoietic cells in the early human embryo. Blood.

[33] Oberlin E, Hafny B, El, Petit-Cocault L, Souyri M (2010). Definitive human and mouse hematopoiesis originates from the embryonic endothelium: a new class of HSCs based on VE-cadherin expression. Development.

[34] Ivanovs A, Rybtsov S, Welch L (2011). Highly potent human hematopoietic stem cells first emerge in the intraembryonic aorta-gonad-mesonephros region. J Exp Med.

[35] North T, Gu TL, Stacy T (1999). Cbfa2 is required for the formation of intra-aortic hematopoietic clusters. Development.

[36] Yokomizo T, Dzierzak E (2010). Three-dimensional cartography of hematopoietic clusters in the vasculature of whole mouse embryos. Development.

[37] Batsivari A, Rybtsov S, Souilhol C (2017). Understanding hematopoietic stem cell development through functional correlation of their proliferative status with the intra-aortic cluster architecture. Stem Cell Rep.

[38] Laurenti E, Göttgens B (2018). From haematopoietic stem cells to complex differentiation landscapes. Nature.

[39] Rybtsov S, Ivanovs A, Zhao S, Medvinsky A (2016). Concealed expansion of immature precursors underpins acute burst of adult HSC activity in foetal liver. Development.

[40] Oberlin E, Tavian M, Blazsek I, Péault B (2002). Blood-forming potential of vascular endothelium in the human embryo. Development.

[41] Sinka L, Biasch K, Khazaal I (2012). Angiotensin-converting enzyme (CD143) specifies emerging lympho-hematopoietic progenitors in the human embryo. Blood.

[42] Kennedy M, Awong G, Sturgeon CM (2012). T Lymphocyte potential marks the emergence of definitive hematopoietic progenitors in human pluripotent stem cell differentiation cultures. Cell Rep.

[43] Ditadi A, Sturgeon CM, Tober J (2015). Human definitive haemogenic endothelium and arterial vascular endothelium represent distinct lineages. Nat Cell Biol.

[44] Canu G, Athanasiadis E, Grandy RA (2020). Analysis of endothelial-to-haematopoietic transition at the single cell level identifies cell cycle regulation as a driver of differentiation. Genom Biol.

[45] Ottersbach K, Dzierzak E (2005). The murine placenta contains hematopoietic stem cells within the vascular labyrinth region. Dev Cell.

[46] Robin C, Bollerot K, Mendes S (2009). Human placenta is a potent hematopoietic niche containing hematopoietic stem and progenitor cells throughout development. Cell Stem Cell.

[47] Li Z, Lan Y, He W (2012). Mouse embryonic head as a site for hematopoietic stem cell development. Cell Stem Cell.

[48] de Bruijn MFTR, Speck NA, Peeters MCE, Dzierzak E (2000). Definitive hematopoietic stem cells first develop within the major arterial regions of the mouse embryo. EMBO J.

[49] Röszer T (2018). Understanding the biology of self-renewing macrophages. Cells.

[50] Calderon B, Carrero JA, Ferris ST (2015). The pancreas anatomy conditions the origin and properties of resident macrophages. J Exp Med.

[51] BainCC Hawley CA, Garner H (2016). Long-lived self-renewing bonemarrow-derived macrophages displace embryo-derived cells to inhabit adultserous cavities. Nat Commun.

[52] Misharin AV, Morales-Nebreda L, Reyfman PA (2017). Monocyte-derived alveolar macrophages drive lung fibrosis and persist in the lung over the life span. J Exp Med.

[53] Ginhoux F, Schultze JL, Murray PJ (2016). New insights into the multidimensional concept of macrophage ontogeny, activation and function. Nat Immunol.

[54] Devisscher L, Scott CL, Lefere S (2017). Non-alcoholic steatohepatitis induces transient changes within the liver macrophage pool. Cell Immunol.

[55] Cai Z, De Bruijn M, Ma X (2000). Haploinsufficiency of AML1 affects the temporal and spatial generation of hematopoietic stem cells in the mouse embryo. Immunity.

[56] Chen MJ, Yokomizo T, Zeigler BM (2009). Runx1 is required for the endothelial to haematopoietic cell transition but not thereafter. Nature.

[57] Tober J, Yzaguirre AD, Piwarzyk E, Speck NA (2013). Distinct temporal requirements for Runx1 in hematopoietic progenitors and stem cells. Development.

[58] Yzaguirre AD, de Bruijn MFTR, Speck NA (2017). The role of Runx1 in embryonic blood cell formation. Adv Exp Med Biol.

[59] Yokomizo T, Hasegawa K, Ishitobi H (2008). Runx1 is involved in primitive erythropoiesis in the mouse. Blood.

[60] Shivdasanl RA, Mayer EL, Orkin SH (1995). Absence of blood formation in mice lacking the T-cell leukaemia oncoprotein tal-1/SCL. Nature.

[61] Vagapova ER, Spirin PV, Lebedev TD, Prassolov VS (2018). The role of TAL1 in hematopoiesis and leukemogenesis. Acta Naturae.

[62] ChenL Kostadima M, Martens JHA (2014). Transcriptional diversity duringlineage commitment of human blood progenitors. Science.

[63] Tsai FY, Keller G, Kuo FC (1994). An early haematopoietic defect in mice lacking the transcription factor GATA-2. Nature.

[64] Fujiwara Y, Chang AN, Williams AM, Orkin SH (2004). Functional overlap of GATA-1 and GATA-2 in primitive hematopoietic development. Blood.

[65] Esner M, Meilhac SM, Relaix F (2006). Smooth muscle of the dorsal aorta shares a common clonal origin with skeletal muscle of the myotome. Development.

[66] Sato Y (2013). Dorsal aorta formation: separate origins, lateral-to-medial migration, and remodeling. Dev Growth Differ.

[67] Durand C, Robin C, Bollerot K (2007). Embryonic stromal clones reveal developmental regulators of definitive hematopoietic stem cells. Proc Natl Acad Sci USA.

[68] PougetC Peterkin T, Simões FC (2014). FGF signalling restricts haematopoieticstem cell specification via modulation of the BMP pathway. Nat Commun.

[69] SouilholC Gonneau C, Lendinez JG (2016). Inductive interactions mediated byinterplay of asymmetric signalling underlie development of adult haematopoieticstem cells. Nat Commun.

[70] Gering M, Patient R (2005). Hedgehog signaling is required for adult blood stem cell formation in zebrafish embryos. Dev Cell.

[71] Peeters M, Ottersbach K, Bollerot K (2009). Ventral embryonic tissues and Hedgehog proteins induce early AGM hematopoietic stem cell development. Development.

[72] McGarvey AC, Rybtsov S, Souilhol C (2017). A molecular roadmap of the AGM region reveals BMP ER as a novel regulator of HSC maturation. J Exp Med.

[73] Wilkinson RN, Pouget C, Gering M (2009). Hedgehog and Bmp polarize hematopoietic stem cell emergence in the zebrafish dorsal aorta. Dev Cell.

[74] Ruiz-Herguido C, Guiu J, D’Altri T (2012). Hematopoietic stem cell development requires transient Wnt/β-catenin activity. J Exp Med.

[75] Chanda B, Ditadi A, Iscove NN, Keller G (2013). XRetinoic acid signaling is essential for embryonic hematopoietic stem cell development. Cell.

[76] Gama-NortonL Ferrando E, Ruiz-Herguido C (2015). Notch signal strength controlscell fate in the haemogenic endothelium. Nat Commun.

[77] Ibarra-Soria X, Jawaid W, Pijuan-Sala B (2018). Defining murine organogenesis at single-cell resolution reveals a role for the leukotriene pathway in regulating blood progenitor formation. Nat Cell Biol.

[78] Marcelo KL, Sills TM, Coskun S (2013). Hemogenic endothelial cell specification requires c-Kit, notch signaling, and p27-mediated cell-cycle control. Dev Cell.

[79] Bowie MB, McKnight KD, Kent DG (2006). Hematopoietic stem cells proliferate until after birth and show a reversible phase-specific engraftment defect. J Clin Invest.

[80] Kozar K, Ciemerych MA, Rebel VI (2004). Mouse development and cell proliferation in the absence of D-cyclins. Cell.

[81] Malumbres M, Sotillo R, Santamaría D (2004). Mammalian cells cycle without the D-type cyclin-dependent kinases Cdk4 and Cdk6. Cell.

[82] Eliasson P, Jönsson JI (2010). The hematopoietic stem cell niche: low in oxygen but a nice place to be. J Cell Physiol.

[83] Laurenti E, Frelin C, Xie S (2015). CDK6 levels regulate quiescence exit in human hematopoietic stem cells. Cell Stem Cell.

[84] Thomson JA (1998). Embryonic stem cell lines derived from human blastocysts. Science.

[85] Takahashi K, Tanabe K, Ohnuki M (2007). Induction of pluripotent stem cells from adult human fibroblasts by defined factors. Cell.

[86] Vallier L, Touboul T, Brown S (2009). Signaling pathways controlling pluripotency and early cell fate decisions of human induced pluripotent stem cells. Stem Cells.

[87] Slukvin II (2013). Hematopoietic specification from human pluripotent stem cells: current advances and challenges toward de novo generation of hematopoietic stem cells. Blood.

[88] Kaufman DS (2009). Toward clinical therapies using hematopoietic cells derived from human pluripotent stem cells. Blood.

[89] Elcheva I, Brok-Volchanskaya V, Kumar A (2014). Direct induction of haematoendothelial programs in human pluripotent stem cells by transcriptional regulators. Nat Commun.

[90] Easterbrook J, Fidanza A, Forrester LM (2016). Concise review: programming human pluripotent stem cells into blood. Br J Haematol.

[91] Moreau T, Evans AL, Vasquez L (2016). Large-scale production of megakaryocytes from human pluripotent stem cells by chemically defined forward programming. Nat Commun.

[92] Chen G, Gulbranson DR, Hou Z (2011). Chemically defined conditions for human iPSC derivation and culture. Nat Methods.

[93] Wiles MV, Johansson BM (1999). Embryonic stem cell development in a chemically defined medium. Exp Cell Res.

[94] Niwa A, Heike T, Umeda K (2011). A novel Serum-Free monolayer culture for orderly hematopoietic differentiation of human pluripotent cells via mesodermal progenitors. PLoS One.

[95] Faial T, Bernardo AS, Mendjan S (2015). Brachyury and SMAD signalling collaboratively orchestrate distinct mesoderm and endoderm gene regulatory networks in differentiating human embryonic stem cells. Development.

[96] Mendjan S, Mascetti VL, Ortmann D (2014). NANOG and CDX2 pattern distinct subtypes of human mesoderm during exit from pluripotency. Cell Stem Cell.

[97] Murry CE, Keller G (2008). Differentiation of embryonic stem cells to clinically relevant populations: lessons from embryonic development. Cell.

[98] Vallier L, Mendjan S, Brown S (2009). Activin/Nodal signalling maintains pluripotency by controlling Nanog expression. Development.

[99] Tam PPL, Behringer RR (1997). Mouse gastrulation: the formation of a mammalian body plan. Mech Dev.

[100] Bernardo AS, Faial T, Gardner L (2011). BRACHYURY and CDX2 mediate BMP-induced differentiation of human and mouse pluripotent stem cells into embryonic and extraembryonic lineages. Cell Stem Cell.

[101] Smith JR, Vallier L, Lupo G (2008). Inhibition of Activin/Nodal signaling promotes specification of human embryonic stem cells into neuroectoderm. Dev Biol.

[102] Morizane A, Doi D, Kikuchi T (2011). Small-molecule inhibitors of bone morphogenic protein and activin/nodal signals promote highly efficient neural induction from human pluripotent stem cells. J Neurosci Res.

[103] Yokomizo T, Watanabe N, Umemoto T (2019). Hlf marks the developmental pathway for hematopoietic stem cells but not for erythro-myeloid progenitors. J Exp Med.

[104] Bruveris FF, Ng ES, Leitoguinho AR (2020). Human yolk sac-like haematopoiesis generates RUNX1- and GFI1/1B-dependent blood and SOX17-positive endothelium. Development.

[105] Lancrin C, Mazan M, Stefanska M (2012). GFI1 and GFI1B control the loss of endothelial identity of hemogenic endothelium during hematopoietic commitment. Blood.

[106] Thambyrajah R, Patel R, Mazan M (2016). New insights into the regulation by RUNX1 and GFI1(s) proteins of the endothelial to hematopoietic transition generating primordial hematopoietic cells. Cell Cycle.

[107] Thambyrajah R, Mazan M, Patel R (2016). GFI1 proteins orchestrate the emergence of haematopoietic stem cells through recruitment of LSD1. Nat Cell Biol.

[108] Wilson NK, Foster SD, Wang X (2010). Combinatorial transcriptional control in blood stem/progenitor cells: genome-wide analysis of ten major transcriptional regulators. Cell Stem Cell.

[109] Lichtinger M, Ingram R, Hannah R (2012). RUNX1 reshapes the epigenetic landscape at the onset of haematopoiesis. EMBO J.

[110] Org T, Duan D, Ferrari R (2015). Scl binds to primed enhancers in mesoderm to regulate hematopoietic and cardiac fate divergence. EMBO J.

[111] Pauklin S, Vallier L (2013). The cell-cycle state of stem cells determines cell fate propensity. Cell.

[112] Lis R, Karrasch CC, Poulos MG (2017). Conversion of adult endothelium to immunocompetent haematopoietic stem cells. Nature.

[113] Sugimura R, Jha DK, Han A (2017). Haematopoietic stem and progenitor cells from human pluripotent stem cells. Nature.

[114] LaRue AC, Lansford R, Drake CJ (2003). Circulating blood island-derived cells contribute to vasculogenesis in the embryo proper. Dev Biol.

[115] Azzoni E, Conti V, Campana L (2014). Hemogenic endothelium generates mesoangioblasts that contribute to several mesodermal lineages in vivo. Dev.

[116] Hirschi KK, Ingram DA, Yoder MC (2008). Assessing identity, phenotype, and fate of endothelial progenitor cells. Arterioscler Thromb Vasc Biol.

[117] Singhal M, Liu X, Inverso D (2018). Endothelial cell fitness dictates the source of regenerating liver vasculature. J Exp Med.

[118] Mariani SA, Li Z, Rice S (2019). Pro-inflammatory aorta-associated macrophages are involved in embryonic development of hematopoietic stem cells. Immunity.

